# Supplementary material for: i-Propylammonium Lead Chloride Based Perovskite Photocatalysts for Depolymerization of Lignin Under UV Light
Source: Molecules. 2020 Jul 31;25(15):3520. doi: 10.3390/molecules25153520 (PMC7435779; doi:10.3390/molecules25153520)
Supplement: Supplementary file 1 [file molecules-25-03520-s001.pdf]

i-Propylammonium Lead Chloride Based Perovskite Photocatalysts for Depolymerization of Lignin Under UV Light

Samia Kausar [1], Ataf Ali Altaf [1,2,*], Muhammad Hamayun [1], Nasir Rasool [3], Mahwish Hadait [1], Arusa Akhtar [1], Shabbir Muhammad [4,5], Amin Badshah [6], Syed Adnan Ali Shah [7,8] and Zainul Amiruddin Zakaria [9,10,*]

Supplemental Data

Supplemental Data

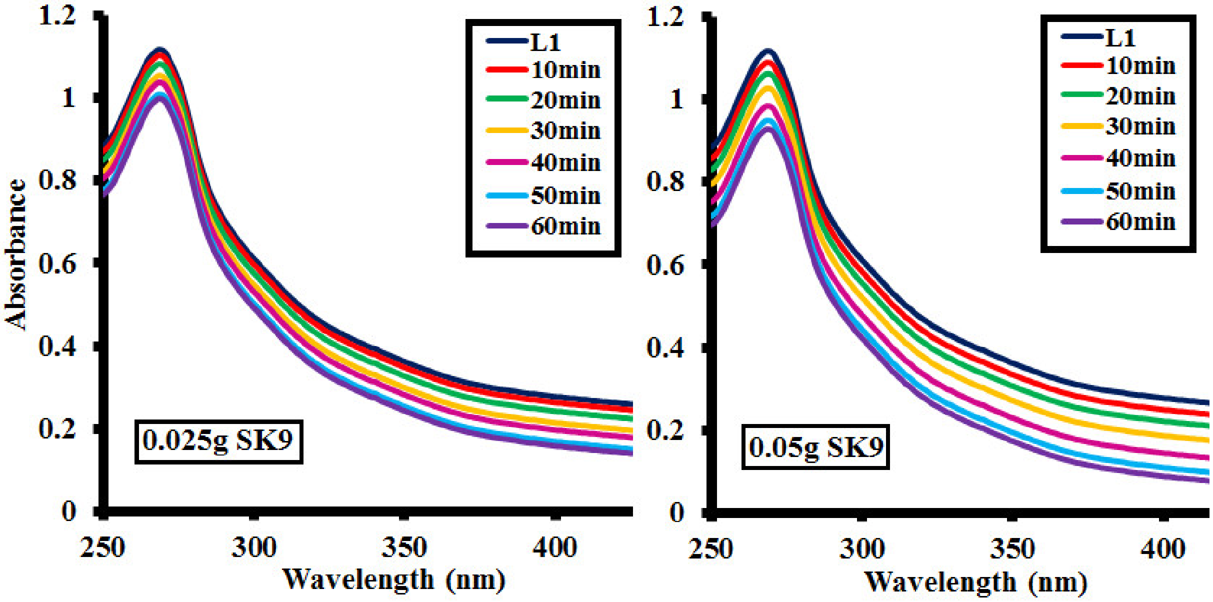

<u>Effect of Catalyst Doses at Different Time Intervals</u>

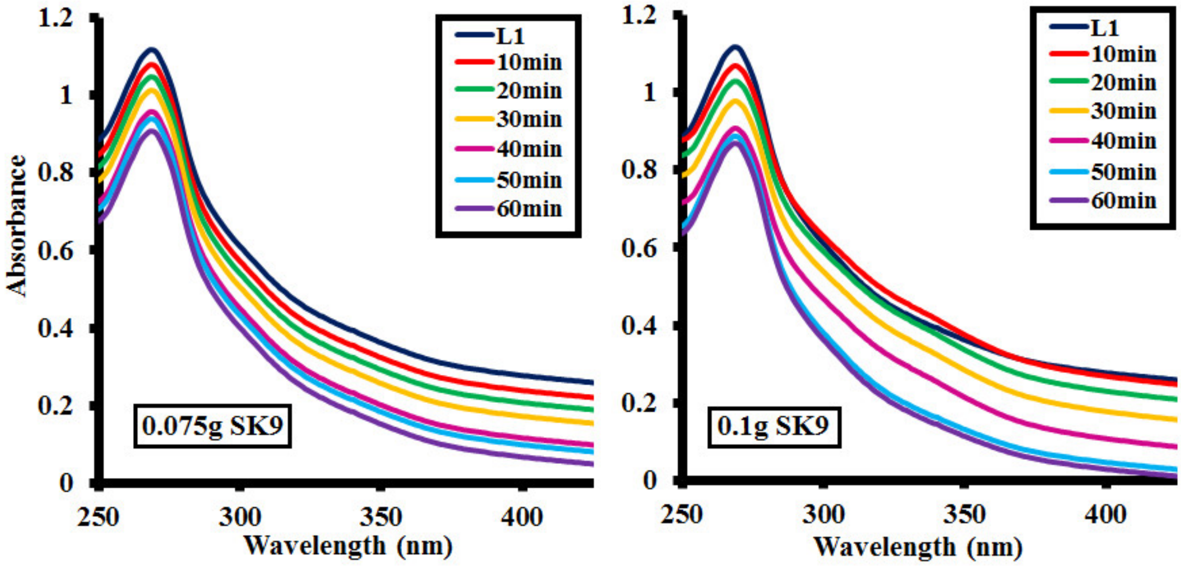

Figure S1: Photocatalytic Depolymerization of Lignin ($L1_{100ppm}$) by different catalyst doses (0.025, 0.05, 0.075 and 0.1g) of **SK9** at different Time Intervals

Effect of Initial Lignin Concentrations at Different Time Intervals

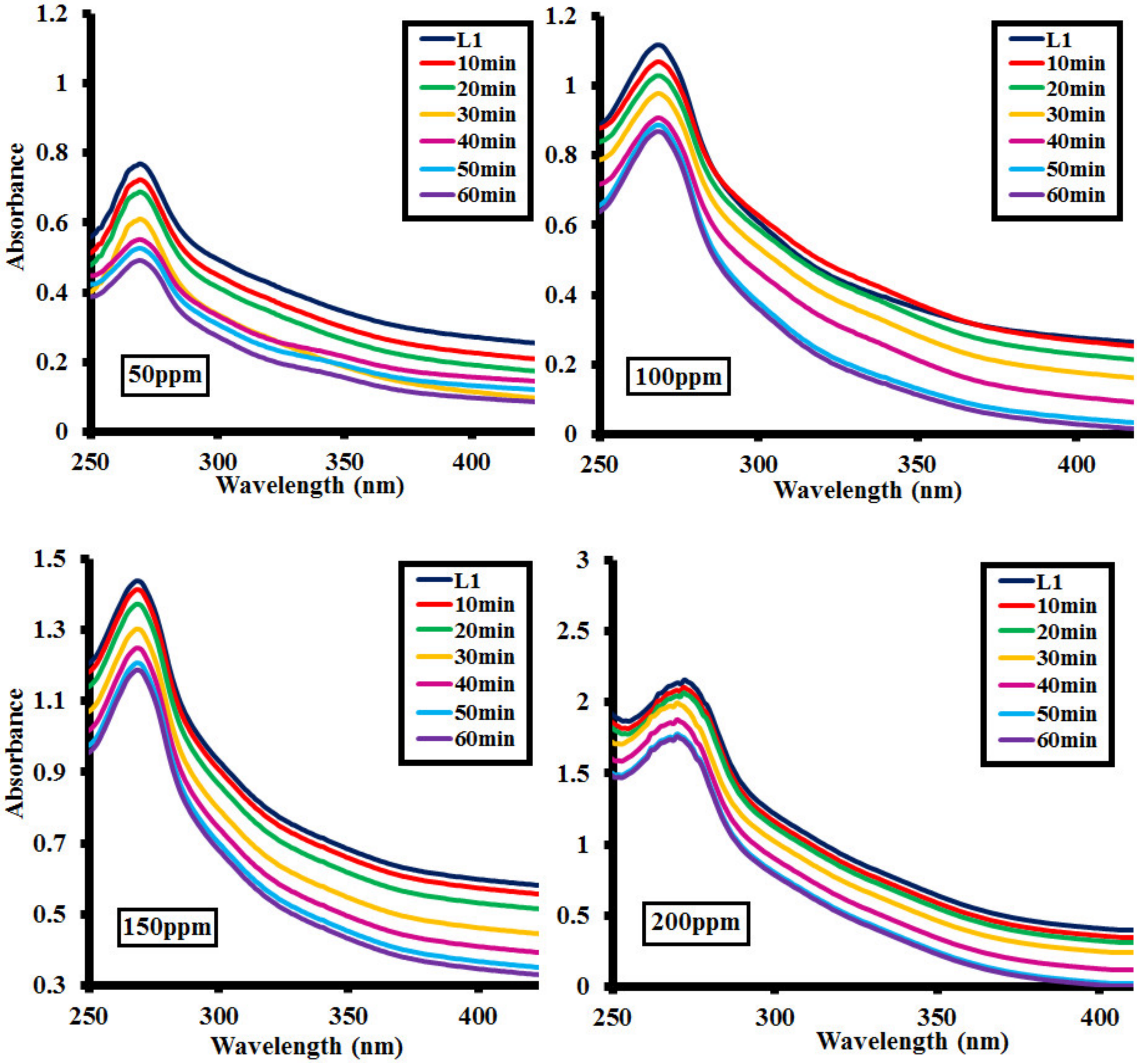

Figure S2: Photocatalytic Depolymerization of Different Initial Lignin Concentrations (50, 100, 150, 200ppm) by **SK9** at different Time Intervals

<u>**Effect of Temperatures at Different Time Intervals**</u>

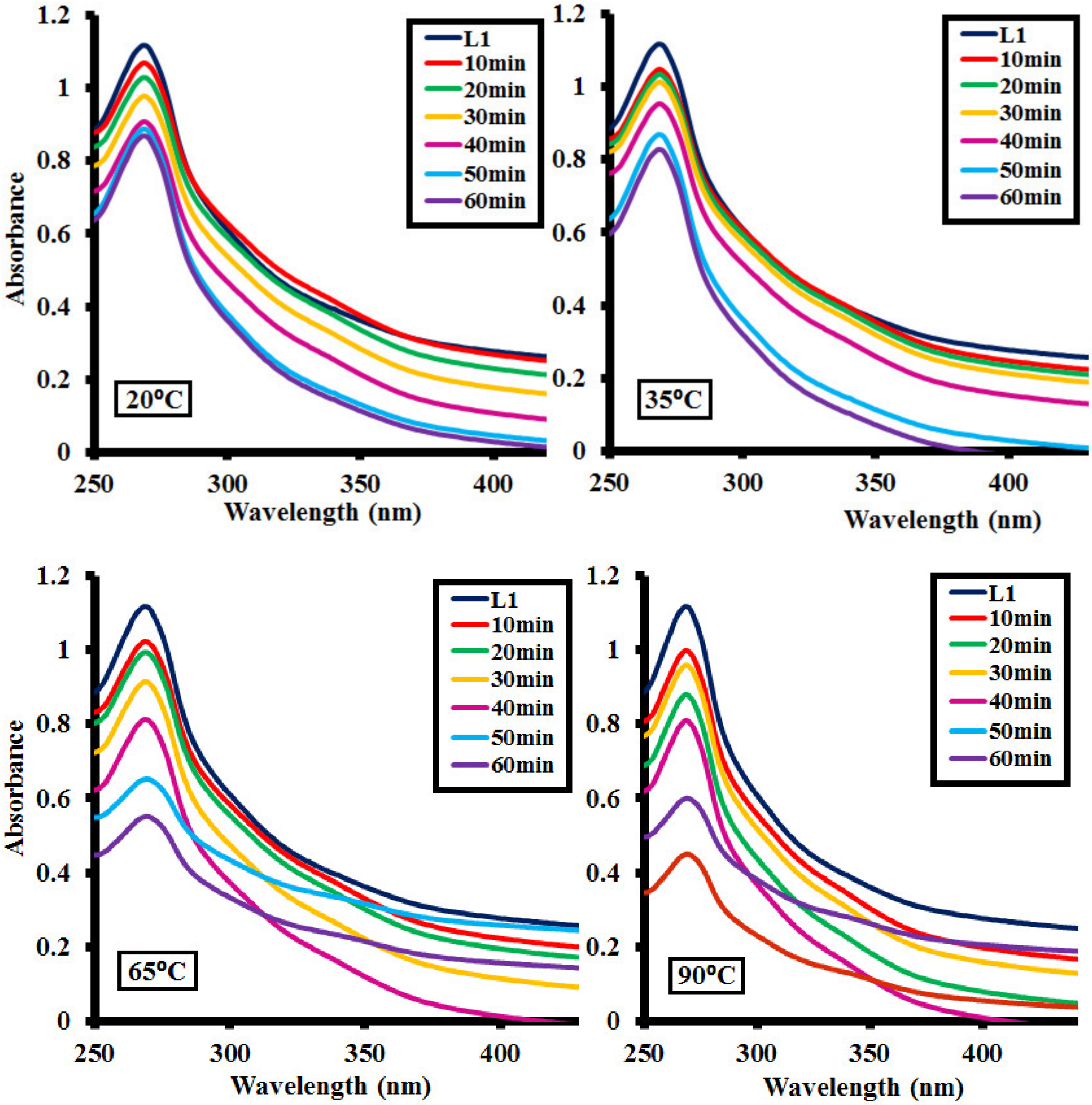

Figure S3: Photocatalytic Depolymerization of Lignin ($L1_{100ppm}$) by **SK9** at varying Temperatures (20, 35, 65, 90°C) at different Time Intervals

S4: GCMS Analysis of Lignin Depolymerization Products L1-SK9 and L1-SK9 (90°C)

MS of Signal 1 from GC (RT = 1.6)

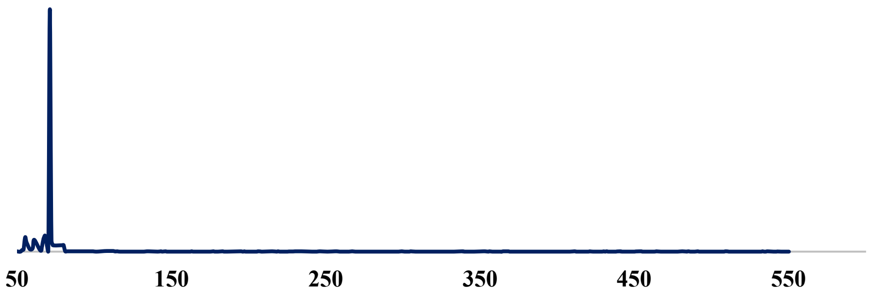

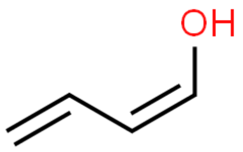

Butadienol

MS of Signal 1' from GC (RT = 1.9)

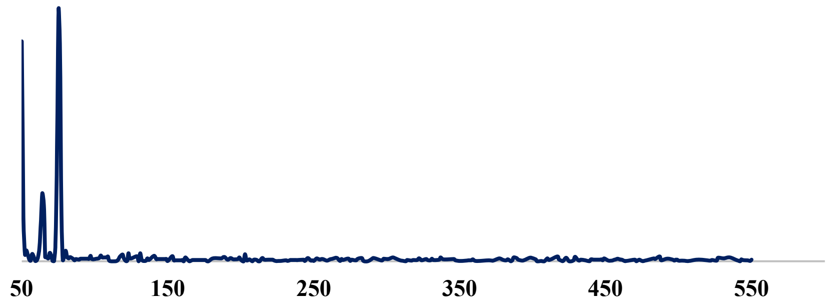

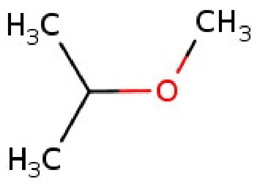

Methoxypropane

MS of Signal 2' from GC (RT = 2.1)

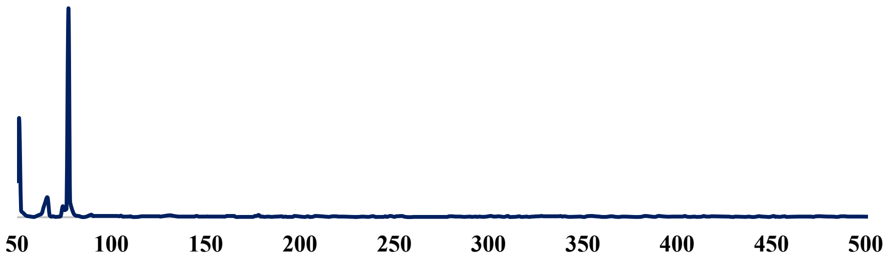

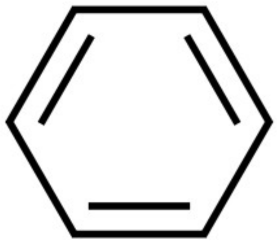

Benzene

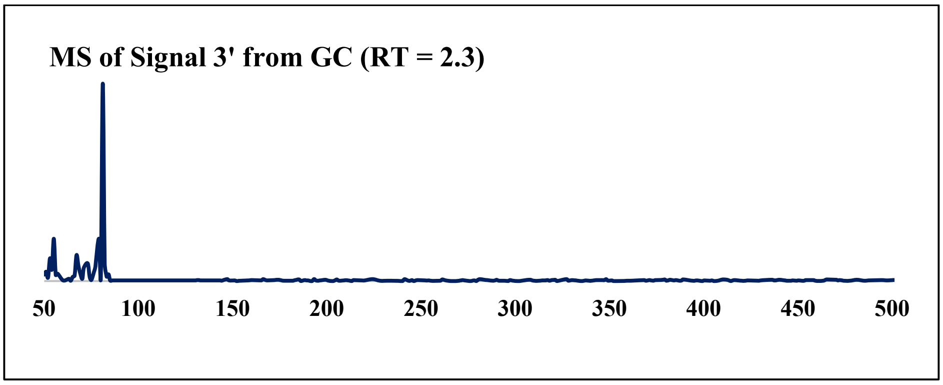

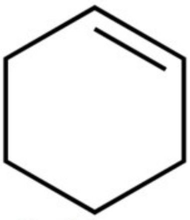

Cyclohexene

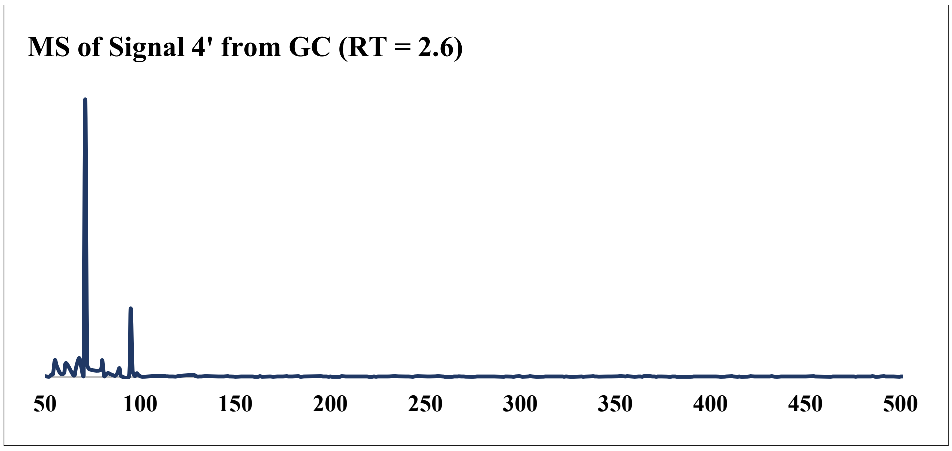

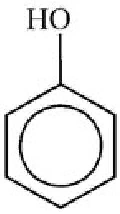

Phenol

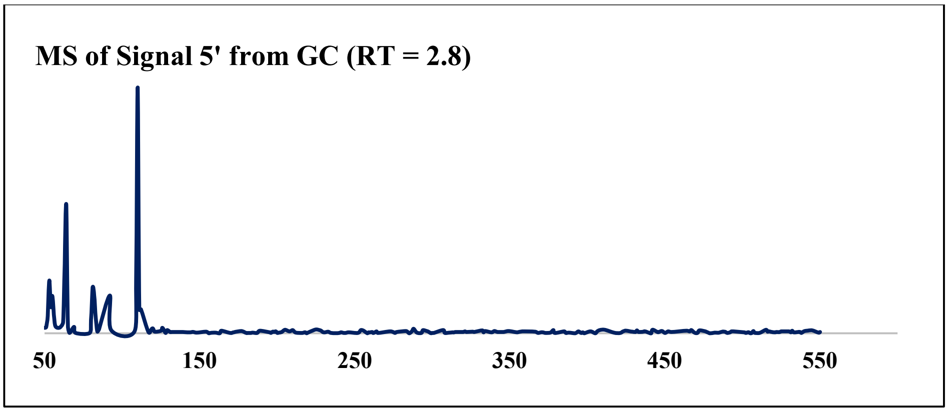

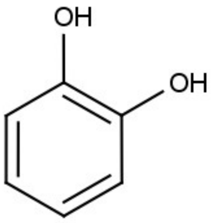

Catechol

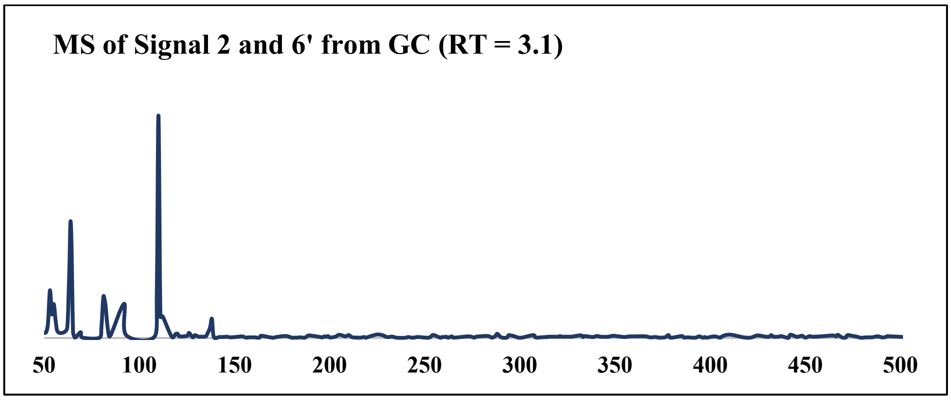

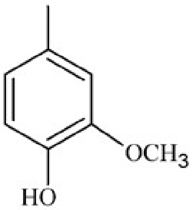

2-methoxy-4-methylphenol

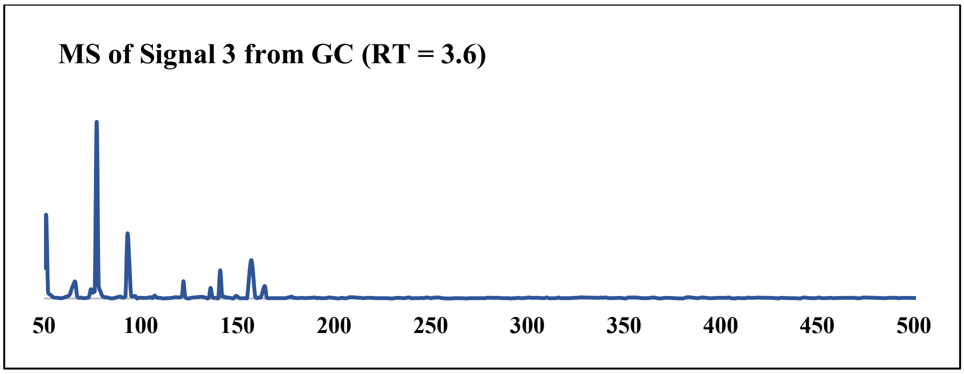

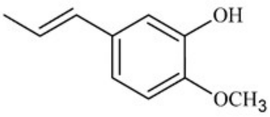

2-methoxy-5-propenyl phenol

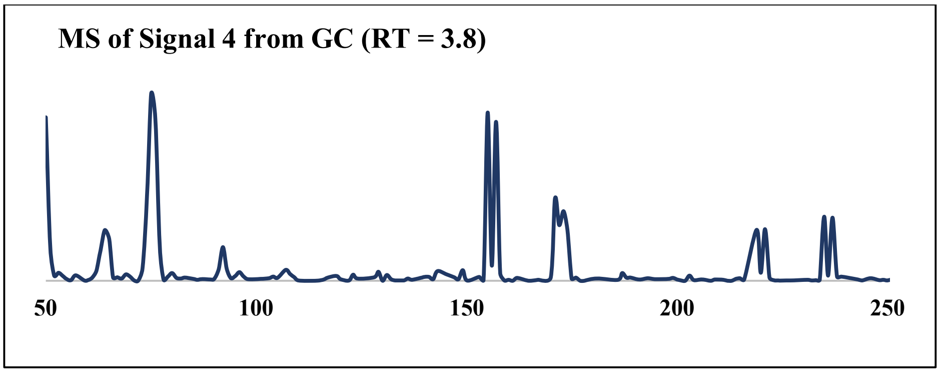

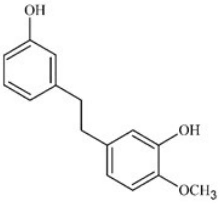

5-[2-(3-hydroxyphenyl)ethyl]-2-methoxyphenol (guaiacyl dimer)
